# Hypertriglyceridemia may contribute to stroke and pancreatitis: A case report and review of the literature

**DOI:** 10.3389/fendo.2022.960343

**Published:** 2022-12-01

**Authors:** Mingyu Zhang, Taoyuan Yin, Feng Xia, Suhong Xia, Wangdong Zhou, Yu Zhang, Xu Han, Kai Zhao, Lina Feng, Ruonan Dong, Dean Tian, Yan Yu, Jiazhi Liao

**Affiliations:** ^1^ Department of Gastroenterology, Tongji Hospital of Tongji Medical College, Huazhong University of Science and Technology, Wuhan, Hubei, China; ^2^ Department of Biliary-Pancreatic Surgery, Tongji Hospital of Tongji Medical College, Huazhong University of Science and Technology, Wuhan, Hubei, China; ^3^ Department of Hepatic Surgery Center, Tongji Hospital of Tongji Medical College, Huazhong University of Science and Technology, Wuhan, Hubei, China

**Keywords:** hypertriglyceridemia, stroke, pancreatitis, metabolic disorders, blood purification

## Abstract

Hypertriglyceridemia (HTG) is one of the most common clinical dyslipidemia. Nevertheless, stroke and acute pancreatitis co-occurrence due to hypertriglyceridemia are extremely rare. We present a case of hypertriglyceridemia-associated stroke and pancreatitis in a 39-year-old woman. The patient’s laboratory tests reported high triglyceride concentrations beyond the instrument’s detection range, and radiological examination showed typical signs of cerebral infarction and acute pancreatitis. The patient received combined blood purification therapy, intravenous thrombolysis with urokinase, and conservative treatment of pancreatitis. We discuss the clinical features, pathogenesis, diagnosis, and treatment of hypertriglyceridemic stroke and pancreatitis combined with the relevant literature. We reviewed the mechanisms by which triglycerides contribute to atherosclerosis and acute pancreatitis. We point out the superiority of combined blood purification therapy and caution physicians about the effects of prescribed drugs on blood lipids.

## Introduction

Severe hypertriglyceridemia is a common risk factor for acute pancreatitis and atherosclerotic vascular disease (1, 2). A population-based study in Copenhagen revealed that triglyceride levels greater than 5 mmol/L were associated with a 3-fold increased risk of ischemic stroke and a 10-fold increased risk of acute pancreatitis compared to levels less than 1 mmol/L (3). The higher the triglyceride level, the higher the risk of suffering from acute pancreatitis. However, very severe and extremely high triglyceride levels may not further increase the risk of stroke (4). It has been mentioned that the co-occurrence of stroke and acute pancreatitis due to hypertriglyceridemia is rare (5). This article detailed such a rare case and searched for similar case reports. We present the first review of two complications of hypertriglyceridemia from the perspectives of clinical features, mechanism, diagnosis, and treatment.

## Case presentation

**Figure 1:**
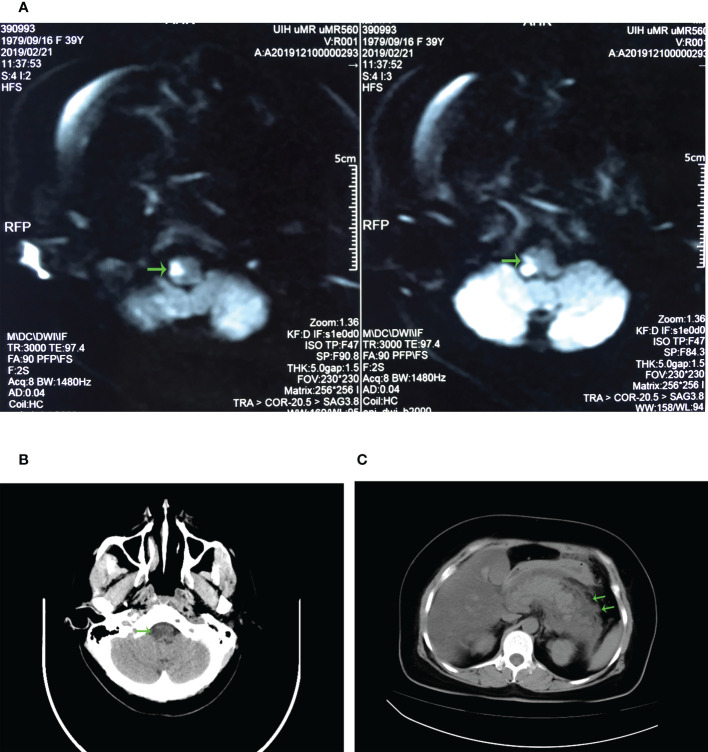
Radiology results. **(A)** Patient’s cranial DWI imaging. The green arrow marks the abnormal signal on the right side of the patient’s medulla oblongata, which is considered as acute cerebral infarction. **(B)** CT imaging of the patient’s head. The green arrow marks a small hypodense lesion on the right side of the medulla oblongata, suggesting the possibility of infarction on the right side of the medulla oblongata. **(C)** CT imaging of the patient’s abdomen. The green arrow marks the swelling and exudation of the pancreas, indicating acute pancreatitis.

A 39-year-old female patient was initially admitted to a local clinic for sudden-onset dizziness, unsteady standing, right-sided limb weakness, and choking cough when swallowing water. The local clinic found that her blood pressure was 180/140mmHg, but a CT head scan showed no significant abnormalities. That evening she experienced the same symptoms above, accompanied by vomiting, and the vomitus was dark green liquid. She was immediately admitted to the emergency department of Qichun County People’s Hospital for head CT. It did not show any significant abnormalities. Given the patient’s symptoms, she underwent further examination. The CDFI revealed left vertebral artery stenosis. Then cranial DWI revealed brainstem infarction (**Figure 1A**), and the patient was given urokinase thrombolysis, oxygen inhalation, antihypertensive, lipid-lowering, and intracranial pressure-lowering (diuretics and mannitol) treatments at once. The patient suddenly developed severe pain in the right abdomen accompanied by vomiting, and then she was taken to our emergency department.

The patient’s acute cerebral infarction was properly treated in Qichun County People’s Hospital, her neurological symptoms were relatively stable, and she was transferred to our hospital mainly because of an abdominal emergency. After admission, relevant laboratory investigations were performed. The patient’s blood pressure was 137/97mmHg, INR was 1.14, APTT value was 30.5s. The patient’s blood pressure and these coagulation parameters were normalized thanks to treatment at other hospital. While the patient’s blood glucose was raised at 16.03 mmol/L, arterial pH was 7.334 with bicarbonate of 17.8 mmol/L, base excess -7.10 mmol/L, and lactate 3.80 mmol/L. Her initial plasma level of the coagulation biomarker D-dimer was 0.87 ug/mL FEU, thrombin-time (TT) was 20.8 seconds, neutrophil count was 18.67 *10^9/L (93.3%), lymphocyte count was 0.62*10^9/L (3.1%), red blood cell count was 5.19*10^12/L, platelet count was 216*10^9/L. C-reactive protein was 50.9 mg/L, pancreatic amylase was 1028 IU/L, lipase was 2509.8 IU/L, total cholesterol was >20.70 mmol/L, triglycerides levels was >50.00 mmol/L, HDL was 0.79mmol/L, LDL was 1.10mmol/L and calcium was 1.52 mmol/L. The hCG value was 2.9 mIU/ml.

CT scans of the head, abdomen and abdominal CT angiography were perfumed in our hospital within 24 hours. At this time, head CT revealed a small hypodense lesion on the right side of the medulla oblongata (**Figure 1B**), which was consistent with the presentation after intravenous thrombolysis of stroke. Abdominal CT revealed pancreatic swelling and exudation (**Figure 1C**), fatty liver, and double renal calculi. Since the patient had symptoms of sudden abdominal pain, we performed an abdominal CTA to investigate whether she had an aortic dissection. No apparent double-lumen structure was observed in the thoracoabdominal aorta.

## Case follow-up

The patient had a history of diabetes, which had not been treated regularly for five years. There was no history of smoking or drinking. Based on the clinical presentation, laboratory tests, and radiological examination, we can determine that the patient successively developed cerebral infarction and acute pancreatitis quickly. We centrally reviewed all laboratory test results, and the patient’s hyperlipidemia, especially the triglyceride level much higher than the reference value, gained our attention. We performed a literature search, we searched Google Scholar for keywords “(cerebral infarction OR embolism OR stroke) AND pancreatitis” and found several cases (**Table 1**). We conclude that it is a rare case, and the patient’s hypertriglyceridemia might cause two complications, cerebral infarction and acute pancreatitis, to co-occur.

**Table 1:** Details of patients with stroke and pancreatitis.

Case	Patient Information	Primary Diagnosis	HTG	Treatment	Outcome
Case 1 Vinod et al (6)	48-year-old male,heavy alcohol consumer	Alcohol-associated pancreatitis, cerebral infarction in the left MCA region	No	Continuous nasogastric aspiration, intravenous fluids, stress ulcer prophylaxis and heparin, antibiotics, mechanical ventilation	Abandon treatment
Case 2 Bhalla et al (7)	25-year-old male,an occasional smoker anda social drinker	Traumatic pancreatitis, multiple infarcts in the left MCA territory	No	Antibiotics, analgesics, jejunostomy feeds and aggressive physiotherapy	Survival
Case 3 Ludwig et al (8)	37-year-old male,hypertension, gastroesophageal reflux disease, pancreatitis, and alcohol abuse	Acute pancreatitis superimposed upon chronic pancreatitis, splenic and renal vein thrombosis, left ICA thrombus, left MCA territory infarction, anemia of chronic disease	No	Thrombolytic therapy, antibiotics, heparin, warfarin, statin, aspirin	Survival
Case 4 Muroi et al (9)	4-year-old male	HL deficiency, an episode of cerebral infarction in the neonatal period and recurrent episodes of hypoglycemia and pancreatitis in childhood	No	Protein restriction and the administration of L-carnitine, complete starvation and administration of nafamostat mesilate	Unknown
Case 5 Patricia et al (10)	37-year-old female,rarely consumed alcohol	Diabetic ketoacidosis, hypertriglyceridemic pancreatitis, bilateral thalamic and extensive pontine cerebellar infarction	Yes20.0 mmol/l	Intravenous fluid resuscitation, insulin and heparin, emergency angioplasty and stent insertion	Abandon treatment
Case 6 Present case	39-year-old female,untreated diabetes	Medulla oblongata infarction, hypertriglyceridemic pancreatitis	Yes>50.00 mmol/L	Intravenous thrombolysis with urokinase, antihypertensive and lipid-lowering treatment, DFPP, HP+CVVH, antibiotics	Survival

Middle cerebral artery (MCA); Internal carotid artery (ICA); 3-hydroxy-3-methylglutaryl-CoA lyase (HL); Double Filtration Plasmapheresis (DFPP); Continuous veno-venous hemofiltration (CVVH); Hemoperfusion (HP).

We want to lower triglyceride levels in patients as early as possible. As the dual filtration plasma exchange has fewer overall adverse effects than the conventional ones, it saves much exogenous plasma and reduces the incidence of blood-borne infections. After obtaining the patient’s consent, we performed dual filtration plasma exchange treatment *via* the right internal jugular vein, using 30 mg of heparin for anticoagulation throughout the procedure, disposing of 3200 ml of plasma, discarding 400 ml of plasma, supplementing with 100 ml of albumin and 300 ml of saline. At the end of the treatment, heparin was neutralized with 50mg of protamine. Due to the patient’s recent history of cerebral infarction and the use of heparin in the dual filtration plasma exchange technique, we closely observed the patient for new bleeding spots throughout the body and were alert for cerebral hemorrhage after cerebral infarction.

As needed, the patient was treated the same day with hemoperfusion combined with continuous veno-venous hemofiltration (CVVH), which has been proven effective in many studies. The whole process took 6 hours, and the ultrafiltration volume was 0.5 liters. The coupled blood purification method reduces triglyceride levels while removing unwanted inflammatory factors, creatinine, blood urea nitrogen, and excess water from the body. Triglyceride level decreased to 4.03 mmol/L after treatment.

**Figure 2:**
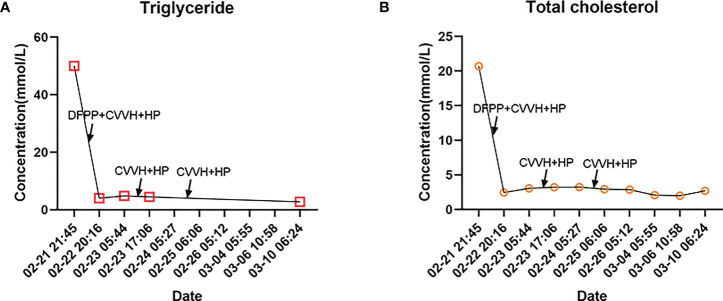
Major lipid trends in our patient. **(A)** Trends in triglyceride changes during hospitalization. Maximum lipid-lowering benefit was obtained in the first combined blood purification therapy, with further reduction in triglyceride levels in the subsequent treatment. **(B)** Trends in total cholesterol changes during hospitalization. The trend was generally consistent with triglyceride levels, and total cholesterol levels returned to normal after the first combined blood purification therapy. Abbreviations: Double Filtration Plasmapheresis (DFPP); Continuous veno-venous hemofiltration (CVVH); Hemoperfusion (HP).

We performed two more hemoperfusions over the next few days combined with CVVH. In addition, we continued to maintain proper fluid balance, use insulin intravenous drip to lower blood sugar, apply of lipid-lowering medication (fibrates), administer antibiotics (biapenem, ornidazole and levofloxacin) to combat infection, inhibit pancreatic enzyme secretion, relieve pain (dizocine and diclofenac sodium), apply of antiplatelet therapy (aspirin and clopidogrel) and correct electrolyte disorders and acid-base balance disorders. We ended up with good laboratory results, we graphed the patient’s major lipid changes (**Figure 2**). The patient was discharged 23 days after admission. At the time of discharge, the patient’s upper and lower limbs were still unable to resist gravity, her NIHSS score was 6.

## Discussion

In **Table 1**, patients in Case 1-3 all developed acute pancreatitis first, either alcohol-related or traumatic. These patients suffered sudden cerebral infarction about a week later, and all three patients had infarcts in the left middle cerebral artery. The cerebral infarction that occurred in these patients was attributed to fat embolism. In patients with severe acute pancreatitis, chylomicrons and very low-density lipoprotein (VLDL) are released from necrotic omental fat into the circulation, in response to the patient’s elevated C-reactive protein, calcium-dependent agglutination of chylomicrons and VLDL occurs, ultimately leading to vascular occlusion (11). When cerebral infarction occurs after acute pancreatitis, and the two conditions are separated by a period of time, and the patient does not have other thrombosis-prone risk factors, we reasonably presume that the cerebral infarction is a rare complication of acute pancreatitis in this case. Case 4 reported a 4-year-old child who developed recurrent acute pancreatitis and acute cerebral infarction. When the patient is very young, we should take genetic defects into account in the first instance. Case 5 and our case both had hyperlipidemia induced by diabetes, especially with triglyceride levels much higher than normal. Patients with hypertriglyceridemia must be alert to the occurrence of pancreatitis and cerebral infarction, and the application of blood purification techniques for rapid lipid lowering seems to be necessary.

Genetic and acquired factors influence triglyceride levels. VLDL and chylomicrons are both major transporters of triglycerides in the body. Some gene defects can lead to defective lipase activity so that triglyceride clearance is inhibited. Other genetic mutations cause reduced hepatic clearance of VLDL and chylomicron remnants. Diabetes, alcoholism, pregnancy and certain drugs can also lead to triglyceride overproduction (12).

Our patient was not tested for the relevant genetic defects. Genetic testing is not a commonly used test at the time of diagnosis. We can only indirectly understand the genetic conditions of patients by asking about family history. More attention should be paid to factors that can cause secondary metabolic disorders, such as poor control of diabetes, heavy drinking and medication use. Our patient did have a history of diabetes that had not been well treated for many years.

Hypertriglyceridemia is the most common cause of acute pancreatitis after biliary system disorders and alcohol (13). As many as 25.6% of acute pancreatitis is associated with dyslipidemia in patients (14).On the one hand, large amounts of triglycerides are hydrolyzed by lipase in the vascular bed of the pancreas to produce excess free fatty acids. Human plasma albumin is limited in amount, and free fatty acids that fail to bind to it aggregate into micellar structures with detergent properties. This micellar structure has toxic effects on platelets, vascular endothelial cells, and acinar cells, eventually inducing ischemia and acidosis. On the other hand, elevated levels of giant lipoproteins rich in triglycerides, namely chylomicrons, increase the viscosity of the blood. A marked rise in the viscosity of the plasma can result in capillary blockage, which exacerbates ischemia and acidosis, and triggers acute pancreatitis through activating trypsinogen (15–17).

LDL cholesterol is a recognized mediator of atherosclerosis, but atherosclerotic vascular events still occur in low LDL cholesterol levels, for which hypertriglyceridemia is a residual risk factor (18). Significant triglyceride increases may be associated with a hypercoagulable state, as factor VIIIc, factor VIIc, fibrinogen, and antithrombin III levels are significantly elevated (19). Hypertriglyceridemia independently promotes increased plasma viscosity and reduced blood flow (20). It has been reported that elevated triglyceride levels are associated with atherosclerotic vascular disease (21, 22). Surprisingly, triglycerides themselves do not directly promote atherosclerotic plaque formation, and in patients with mild to moderate hypertriglyceridemia, VLDL, their residues, and medium-density lipoproteins put individuals at increased risk of atherosclerosis. While in patients with severe hypertriglyceridemia, the cholesterol deposited in plaque may originate from triglyceride-rich chylomicron residues (23). Chylomicron and VLDL remnants are small in diameter and easily penetrate the arterial wall into the subendothelial space (24, 25). These residual particles carry more cholesterol than LDL and can be taken up directly by scavenger receptors on macrophages without oxidative modification, leading to the eventual formation of atherosclerotic plaques (22, 26–28). In addition, free fatty acids generated by triglyceride hydrolysis increase ROS production and induce mitochondrial dysfunction. Mitochondrial dysfunction impaired aerobic capacity, and increased ROS concentrations induce endothelial dysfunction/apoptosis and vascular smooth muscle cell proliferation/apoptosis, also leading to eventual atherosclerotic progression (29). Our patient had relatively low HDL levels, hypertriglyceridemia is usually accompanied by low HDL levels, making it challenging to analyze the effect of a single factor on atherosclerotic vascular events. Numerous Mendelian randomization analyses reveal that elevated levels of residual particulate cholesterol contribute to ischemic heart disease and suggest that HDL cholesterol may not be causally related to the risk of atherosclerotic vascular events (30–32). One study reported that the risk of coronary heart disease due to hypertriglyceridemia was attenuated or even eliminated after adjusting for other risk factors (33). Their data adjustment seems to be biased. They excluded HDL cholesterol and non-HDL cholesterol as confounding factors, whereas non-HDL cholesterol is primarily composed of TG-rich chylomicron remnants and VLDL remnants, with which triglycerides are inextricably linked. This study confirms from another perspective that TG indirectly, but not directly, promotes atheromatous plaque formation.

In the past, lipid disorder was only regarded as a secondary phenomenon of alcoholic or gallstone pancreatitis (34). However, high triglyceride levels are now a risk factor for developing pancreatitis (35). In patients with dyslipidemia, those with acute pancreatitis had higher maximal triglyceride levels than those without pancreatitis. However, there was no correlation between the level of triglycerides and the degree of pancreatitis (36). Controlling triglyceride levels could effectively reduce the risk of recurrence of pancreatitis (37). Moreover, because examination on admission to the hospital is later than the onset of the disease, the initial triglyceride level will be much higher than the laboratory values. Obtaining serum TG levels close to the onset of pain during the initial or recurrent episodes of acute pancreatitis is essential to identifying patients with HTG-induced acute pancreatitis (12). Triglyceride levels should be monitored as early as possible and daily at the onset of acute pancreatitis to avoid unnecessary missed diagnoses or misdiagnoses. Serum triglyceride levels in the first three days of onset are helpful for accurately diagnosing hypertriglyceridemia-induced pancreatitis (14). Triglyceride concentrations > 1000 mg/dl are generally considered to increase the risk of pancreatitis. The risk is even more significant in individuals with very severe hypertriglyceridemia (triglycerides >2000 mg/dl) (12).

Plasmapheresis can rapidly reduce the levels of chylomicrons and triglycerides in the serum. Several case reports and clinical guidelines demonstrated the feasibility of plasmapheresis for the treatment of patients with severe or deteriorating hypertriglyceridemia combined with complications (38–40). Long-term, regular dual filtration plasmapheresis can benefit patients with hypertriglyceridemia and related recurrent complications who are not well treated with conventional dietary therapy and medications (41) Dual filtration plasmapheresis is a technical innovation with high safety, broad applicability, low nutrient loss and low plasma usage. The initial lipid-lowering treatment in our case used this approach. By the way, measuring a patient’s total cholesterol level may predict the efficacy of triglyceride-lowering therapy (42). However, plasmapheresis is expensive and is not without risks. It requires the establishment of intravenous access and is an invasive treatment. It may develop complications related to bacteremia, deep vein thrombosis, and bleeding. It has been shown that there is no significant difference in morbidity and mortality whether patients are treated with plasmapheresis or non-invasive conservative treatment (43). Other studies revealed no difference in triglyceride reduction rates or clinical outcomes between patients who did or did not receive plasmapheresis (44, 45). These observational studies described above could not control for all possible confounding factors. Therefore, we need more rigorous randomized controlled trials to explore the value of plasmapheresis.

CVVH is a renal replacement therapy, a hemodialysis technique used to correct metabolic disorders and remove inflammatory mediators and toxic substances from patients in critical care (46). Successful treatment of hyperlipidemia-associated acute pancreatitis by CVVH has been reported (47). Another case reported hyperlipidemia and other serious side effects in children induced by propofol, a sedative drug, and CVVH resolved these abnormalities (48). A randomized controlled trial suggested that a subgroup classification of patients was needed to discuss the clinical benefits of CVVH for them. The efficacy of CVVH treatment is significant in patients with intra-abdominal pressure ≥20 mm Hg. However, the efficacy of CVVH in patients with intra-abdominal pressure <20 mm Hg is unclear (49). Serum IL-17 is somewhat predictive of the clinical benefit of CVVH in patients with severe pancreatitis (50).

Various combinations of blood purification techniques are commonly used in clinical practice. Hemoperfusion combined with CVVH significantly reduces triglyceride levels and removes excess inflammatory factors from the internal environment, significantly improving the prognosis of patients (51). In patients with hyperlipidemic severe acute pancreatitis, a prospective controlled study showed that hemoperfusion plus high-volume hemofiltration significantly improved serological parameters and clinical manifestations (52). The treatment, in our case, draws on their experience. In addition, an observational study reported that the use of plasma exchange combined with CVVH, a sequential hemodialysis modality, was effective in treating patients with hyperlipidemia-induced acute pancreatitis in a critical care setting (53).

We can use some medications to lower triglyceride levels, such as fibrates, nicotinic acid, and omega-3 fatty acids/fish oil (12, 54, 55), and drug combinations may be more beneficial than single drugs. Previous studies have reported the occurrence of rhabdomyolysis, a malignant event caused by a combination of fibrates and statins (56). In the latest study, the safety profile of fenofibrate in combination with a statin was acceptable, and this combination significantly reduced the risk of vascular events in patients with typical diabetic dyslipidemia (57). In recent years, researchers have made significant progress in developing targeted therapies, such as volanesorsen (58, 59), evinacumab (60, 61), lomitapide (62) and icosapent ethyl (63).

Diabetes is one of the most common acquired factors causing hypertriglyceridemia. Diabetes can independently increase a patient’s risk of developing acute pancreatitis after controlling for other risk factors (64). Insulin effectively increases the peripheral activity of lipoprotein lipase while controlling blood glucose and reverses the effects of insulin resistance on the liver (12).

It has been shown that continuous intravenous administration of heparin to patients leads to a decrease in lipoprotein lipase activity, which may ultimately lead to the accumulation of chylomicrons in the circulation (65). Therefore, the use of heparin is controversial. More experiments are needed to investigate whether it can be used or find the recommended dose.

It is well documented that chronic heavy alcohol consumption leads to a significant increase in triglyceride levels (66). Abstinence from alcohol and sugar, a low-fat diet, and enhanced physical exercise have been written into the treatment algorithm of an Australian hospital (67). Monitoring for distant complications associated with this acute event, such as chronic pancreatitis or pancreatic pseudocysts, will continue to be required (68), so regular follow-up is advisable.

During our literature search, we unexpectedly found a variety of prescribed medications that can cause a spike in triglyceride levels in patients, which must be taken into account by clinicians. The relevant drugs are summarized in the table (**Table 2**).

**Table 2:** Drug-induced dyslipidemia.

Drug	Function	Effect on TG levels	Outcome	References
Tamoxifen	A non-steroidal estrogen antagonist widely used in adjuvant hormonal therapy for breast cancer.	11.8 g/L	A diagnosis of acute pancreatitis grade E was made,the recurrence of pancreatitis when the drug was readministered.	(69)
		2569 mg/dL	Acute pancreatitis, thoracic ascites, and gastrointestinal tract dilatation.	(70)
Oestradiol	A high-dose oral oestrogen therapy after feminising genital reconstructive surgery.	72mmol/L	Acute pancreatitis, the liver showed a diffuse increase in echogenicity in keeping with fatty change, development of a pancreatic pseudocyst	(71)
		>7000 mg/dL	Fevers with tachycardia, acute severe pancreatitis, bibasilar pneumonitis with ascites and retroperitoneal fluid	(72)
Prednisone	A corticosteroid for the treatment of urticarial rash	49.0 mmol/L	Acute uncomplicated pancreatitis	(73)
	A corticosteroid for the treatment of chronic obstructive pulmonary disease exacerbation	72.4 mmol/L	Acute-onset pancreatitis	(73)
Isotretinoin	A retinoid derivative for severe acne	1300 mg/dL	Low-grade fever, mild pancreatitis	(74)
		61 mg/dl	MRCP of the abdomen showed inflammation of the pancreas and mild ascites.	(75)
Adalimumab	Treatment of psoriasis	4425 mg/dl	Acute pancreatitis	(76)
Capecitabine	A prodrug of the cytotoxic agent 5-fluorouracil used in the chemotherapy treatment of adenocarcinoma of the colon	2126 mg/dL	Hypertriglyceridemia only	(77)
Brentuximab	An antibody-drug conjugate for relapsed T-cell lymphoma with CD30 expression	3175 mg/dL	Acute interstitial pancreatitis and hepatic steatosis, Suspected pseudocyst	(78)
Metoprolol	A beta-blockade for peripheral and coronary artery disease	4956 mg/dL	Acute pancreatitis, hypotension.	(79)
Propofol	Sedation	930 mg/dL	Hypertriglyceridemia leads to CRRT circuit malfunction	(80)

This case has many limitations in the diagnostic work-up and treatment options for the patient’s neurological symptoms. We lacked some outcome measures that are important for the prognosis of stroke patients. Before the patient was transferred to our hospital, cerebral angiography, magnetic resonance angiography and transcranial Doppler could not be performed due to the local hospital’s lack of medical equipment and the patient’s financial constraints. After admission, the patient’s neurological symptoms were stable, while the abdominal symptoms were severe. On the one hand, we were unable to wean the patient off the ventilator, and on the other hand, the patient had limited economic conditions. So we did not perform further magnetic resonance spectrum, we performed general care for the patient after thrombolysis and focused our treatment on the management of severe acute pancreatitis.

## Conclusion

In addition to LDL, the main driver of atherosclerosis, high levels of triglycerides also need to be taken seriously by the public for their potential risk of severe vascular events. The diagnosis of dyslipidemia at the beginning of the disease is crucial. It is also important to use blood purification techniques to reduce triglyceride levels as early as possible. Multiple coupled hemodialysis techniques may be superior to single techniques. Genetic analysis can be performed for those patients with poor response to conventional lipid-lowering therapy, patients with familial dyslipidemia, or patients with recurrent hyperlipidemic complications. Once genetic defects are found, we can apply biological agents to target the disease. Hospitalized patients often have multiple comorbidities, and physicians must be aware of the side effects of different medications when prescribing.

## Data availability statement

The original contributions presented in the study are included in the article/supplementary material. Further inquiries can be directed to the corresponding authors.

## Ethics statement

Written informed consent was obtained from the individual(s) for the publication of any potentially identifiable images or data included in this article.

## Author contributions

MZ, TY and FX contributed to the conception and design of the manuscript. SX, WZ, YZ, XH and KZ searched databases for information on the hypertriglyceridemia. MZ, LF and RD wrote the first draft of the manuscript. DT, YY and JL revised the primary manuscript. YY and JL provided funds for open access. All authors contributed to the article and approved the submitted version.

## Funding

This work is supported by the National Natural Science Foundation of China NO:81900476(YY), No. 81672392 (JL); the National Key R&D Program of China (2021YFC2600203).

## Conflict of interest

The authors declare that the research was conducted in the absence of any commercial or financial relationships that could be construed as a potential conflict of interest.

## Publisher’s note

All claims expressed in this article are solely those of the authors and do not necessarily represent those of their affiliated organizations, or those of the publisher, the editors and the reviewers. Any product that may be evaluated in this article, or claim that may be made by its manufacturer, is not guaranteed or endorsed by the publisher.
